# Working memory, cortical dopamine tone, and frontoparietal brain recruitment in post-traumatic stress disorder: a randomized controlled trial

**DOI:** 10.1038/s41398-021-01512-6

**Published:** 2021-07-12

**Authors:** Andrew J. Westphal, Michael E. Ballard, Nicholas Rodriguez, Taylor A. Vega, Mark D’Esposito, Andrew S. Kayser

**Affiliations:** 1 Department of Neurology, University of California, San Francisco, San Francisco, CA USA; 2 Division of Neurology, VA Northern California Health Care System, Martinez, CA USA; 3 Helen Wills Neuroscience Institute, University of California, Berkeley, Berkeley, CA USA

**Keywords:** Human behaviour, Clinical pharmacology, Learning and memory, Psychiatric disorders

## Abstract

Post-traumatic stress disorder (PTSD) leads to impairments in both cognitive and affective functioning. Animal work suggests that chronic stress reduces dopamine tone, and both animal and human studies argue that changes in dopamine tone influence working memory, a core executive function. These findings give rise to the hypothesis that increasing cortical dopamine tone in individuals with greater PTSD symptomatology should improve working memory performance. In this pharmacological functional magnetic resonance imaging (fMRI) study, 30 US military veterans exhibiting a range of PTSD severity completed an emotional working memory task. Each subject received both placebo and the catechol-O-methyl transferase inhibitor tolcapone, which increases cortical dopamine tone, in randomized, double-blind, counterbalanced fashion. Mnemonic discriminability (calculated with d′, an index of the detectability of working memory signals) and response bias were evaluated in the context of task-related brain activations. Subjects with more severe PTSD showed both greater tolcapone-mediated improvements in d′ and larger tolcapone-mediated reductions in liberally-biased responding for fearful stimuli. FMRI revealed that tolcapone augmented activity within bilateral frontoparietal control regions during the decision phase of the task. Specifically, tolcapone increased cortical responses to fearful relative to neutral stimuli in higher severity PTSD subjects, and reduced cortical responses to fearful stimuli for lower severity PTSD subjects. Moreover, tolcapone modulated prefrontal connectivity with areas overlapping the default mode network. These findings suggest that enhancing cortical dopamine tone may represent an approach to remediating cognitive and affective dysfunction in individuals with more severe PTSD symptoms.

## Introduction

Post-traumatic stress disorder (PTSD) is a potentially debilitating mental disorder that affects up to 22.1% of US military veterans [1]. It has been associated with difficulties in both executive and affective functioning, and individuals with PTSD report that working memory is a particularly significant domain of impairment [2]. Given the central role of dopamine within prefrontal cortex (PFC) for working memory in both animals and humans [3, 4], it has been argued that this PTSD-related impairment may be linked to dysfunctional dopaminergic neurotransmission in PFC [5]. In support of this idea, chronic stress in rodents results in tonically low levels of prefrontal dopamine and corresponding deficits in spatial working memory that can be reversed by the administration of a D1 dopamine agonist [6]. In parallel, other rodent models demonstrate that dopamine is released within PFC and limbic regions when emotional stimuli are processed [7]. In particular, D1 receptor activation has been shown to be important for emotion regulation, as D1-deficient mice exhibit normal acquisition but impaired extinction of fear memories, and infusions of a D1 agonist into the rodent PFC block emotional memory recall [8].

This link between executive and affective impairments is similarly consistent with the identification of higher-order brain regions important for emotion regulation and working memory in humans. In addition to hyperactive responses to emotional stimuli in limbic regions including the amygdala, functional neuroimaging of anxiety disorders has identified extensive hypoactive responding in areas thought to support emotion regulation, including the anterior cingulate and medial PFC [9–12]; and a series of studies has shown that dopaminergic processes in these regions are critical for fear extinction for a review, see: [5, 13]. Likewise, individuals with PTSD show altered neural responses within frontoparietal networks during working memory tasks [14, 15], and reduced activity can be seen in lateral PFC both during retrieval [14] and in subjects with more severe symptoms [16]. Thus, dopamine-related deficits that prevent fear extinction may also impair working memory in subjects with PTSD, rendering them particularly susceptible to working memory failures when memoranda include emotional content.

Here we aimed to determine whether working memory performance could be improved in subjects with PTSD via the administration of tolcapone, a medication that inhibits the degradation of dopamine by catechol-O-methyl transferase (COMT), and therefore augments cortical dopamine tone in response to local depolarization [17]. Because the cortex, unlike the striatum, is reliant upon COMT for the removal of ~50% of released dopamine [18], and because cortical regions including PFC are rich in D1 receptors compared to other dopamine receptor subtypes [19], this inhibition of COMT is also thought to induce a relatively selective upregulation of cortical D1 activity [20]. Similar studies in rodents suggest that these cortical effects might be particularly prominent in the prefrontal cortex [18, 21, 22]. In keeping with these ideas, research in humans has shown that the expression of COMT mRNA is comparatively greater in the prefrontal cortex than in the striatum [23]. Furthermore, dopamine transporter profiling in monkeys suggests that, compared to the striatum, frontoparietal regions associated with working memory processes [24] generally express lower dopamine transporter densities, with transporters situated further from the synapse and therefore less likely to contribute to the termination of dopaminergic actions [25].

In behavioral work in humans, tolcapone has demonstrated effects that likewise correlate with cortical, primarily prefrontal, BOLD changes. A pioneering study that administered tolcapone while subjects performed the N-back, a canonical working memory task, showed improvements in working memory processing, primarily in subjects with presumptively lower dopamine tone (as assessed by COMT genotype); and corresponding BOLD effects were interpreted as improved neural efficiency in lateral prefrontal cortex [26]. More recent data has shown that tolcapone might improve maintenance processes in visual working memory circuits [27]. Similarly, tolcapone has demonstrated significant effects on affective-motivational behaviors. Reduced discounting of delayed rewards on tolcapone has been found to vary with baseline impulsivity in both control [28] and patient [29] populations, though inversely in the former case, and directly in the latter. In both studies, anterior cortical regions (the anterior insula and the inferior frontal gyrus) correlated with these changes. Together these findings are consistent with work in both monkeys [3] and humans [4] demonstrating that the relationship between dopamine and performance exhibits an inverted-U relationship, and that working memory and affective-motivational functions are both impacted by changes in cortical dopamine tone.

Given these properties, we hypothesized that if greater PTSD symptomatology correlates with lower cortical dopamine tone, tolcapone would more effectively improve working memory in individuals with more severe PTSD symptoms. By implication, we also hypothesized that tolcapone might decrease performance in subjects with low PTSD severity, assuming that dopamine tone may already be optimal for these subjects. Furthermore, given its influence on affective-motivational circuits, we predicted that tolcapone would prove especially beneficial to higher severity PTSD subjects for emotionally arousing stimuli. Last, we predicted that these behavioral improvements would be associated with improved recruitment of frontoparietal networks supporting working memory and emotion regulation.

## Materials and methods

### Participants

Fifty-five military veterans were recruited for this study (NCT #02260570, available at https://clinicaltrials.gov) from outpatient clinics within the United States Department of Veteran Affairs (VA) Northern California, Palo Alto, and San Francisco Health Care Systems. Data were collected at the University of California, San Francisco (UCSF) Research Clinic located at the Henry H. Wheeler Jr. Brain Imaging Center on the University of California, Berkeley (UCB) campus. Written informed consent was obtained in accordance with both VA and University of California Institutional Review Board procedures. All research subjects underwent a history and physical exam, including blood testing to assess liver function, in order to identify potential contraindications to tolcapone use or MRI scanning (see the Supplementary Methods for a full list of the inclusion and exclusion criteria). Twenty-five of 55 subjects were excluded from the study: 17 participants were deemed ineligible at screening based on inclusion and exclusion criteria, 3 subjects withdrew from the study prior to being allocated to a drug intervention, 3 participants withdrew from the study after being allocated to a drug intervention, and 2 participants were excluded due to technical issues during study procedures (Supplementary Figure 1). PTSD severity was assessed with the Clinician-Administered PTSD Scale for the DSM-5 (CAPS-5) [30]. For a histogram of PTSD severities for participants in this study, see Supplementary Figure 2. The 30 participants (5 female) who completed the study had a mean age of 35.5 ± 8.3 (SD) years (range 22–49; see Supplementary Table 1 for additional demographic information). Participants were compensated for their participation: they received a total of ~$325 for completing the study, including $12 per hour for behavioral testing, $20 per hour for MRI scanning, and a $100 completion bonus for participating in all study procedures.

### Experimental procedure

In the randomized, double-blind, crossover design, each subject received either placebo or a single 200 mg dose of tolcapone during the first MRI session, and the other treatment during the second session. The dose was chosen based on our previous studies showing that 200 mg of tolcapone leads to significant behavioral effects [28, 29, 31]. The allocation of drug order was determined by a researcher unaffiliated with the study (Dr. Jennifer M. Mitchell, UCSF) using a random number generator (https://random.org); thus, all other researchers were blinded to drug identity. Drug order was counterbalanced across participants. Because tolcapone can discolor the urine, 25 mg of the B-vitamin riboflavin was added to both tolcapone and placebo in order to mask this effect and to prevent inadvertent subject unblinding. Participants reported no potential side effects and were ultimately unable to correctly identify whether they received medication or placebo after each MRI session: accuracy was 34.5% for the first MRI session (10/29 guesses correct with one missing data point; *p* = 0.14, binomial theorem) and 50% for the second MRI session (15/30 guesses correct, *p* = 0.86).

**Fig. 1:**
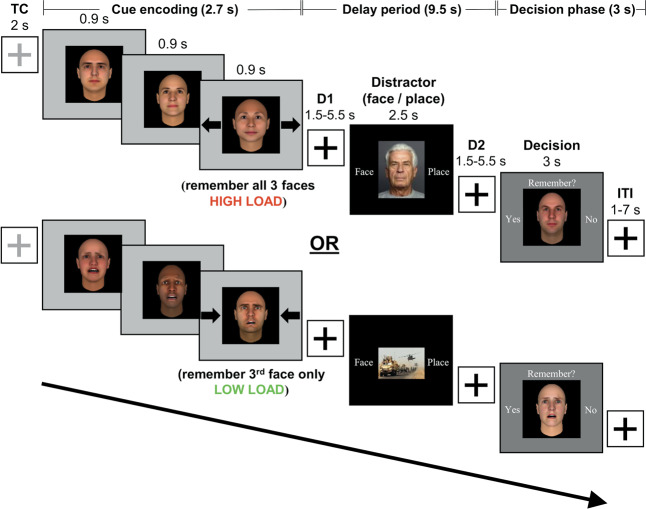
Schematic diagram of the working memory task. During encoding, participants viewed three faces (cues) presented sequentially over 2.7 s. Upon the presentation of the third face, participants maintained either all three faces (high load, depicted in top row—i.e., arrows pointing outward) or the last face only (low load, depicted in bottom row—i.e., arrows pointing inward). After a jittered delay (D1) lasting 1.5, 3.5, or 5.5 s, subjects saw a distractor image for 2.5 s, during which time they made a button press to indicate whether the photograph showed a face or a place. After a second jittered delay (D2), subjects were presented with another face (decision) for 3 s, at which time they indicated whether they remembered it from the encoding period. A fixation cross was then displayed for the 1–7 s inter-trial interval (ITI) that preceded the next trial.

Participants were briefly trained on the working memory task prior to each MRI session to ensure that the participants were familiar with the task instructions. Sixty minutes after ingesting study drug, participants entered the MRI scanner for the collection of task fMRI data and structural images. This timing ensured that acquisition of fMRI data was centered about the time of peak tolcapone concentration (120 min, tolcapone package insert, Valeant Pharmaceuticals). For each fMRI session, participants completed 160 trials of a working memory task including affective conditions and a distractor (Fig. 1). Trials began with the presentation of the 2 s trial cue (a light gray cross on a white background) followed by a 2.7 s cue encoding period during which 3 novel, computer-generated, male and female face stimuli (FaceGen Modeller v3.5; Singular Inversions, Inc., Toronto, Ontario, Canada) expressing either neutral or fearful affect were sequentially presented for 0.9 s each (see also Supplementary Methods). Accompanying the third face cue presentation were black arrows on either side of the image; they pointed either inward to signify that only the last face should be remembered (low load condition) or outward to indicate that all 3 faces should be remembered (high load condition) throughout the following 9.5 s delay period. During the delay, a distractor image was displayed for 2.5 s: a photograph of a face that was either affectively neutral or fearful (per the FACES database, Max Planck Institute, with permission), or a scene that was affectively neutral or arousing (public domain neutral and military-related scenes, respectively, collected by author M.E.B. and validated in pilot behavioral testing). To ensure that participants attended to the distractor, they were required to indicate with a button press whether the distractor image was a “Face” or “Place”. During the decision phase, a face stimulus within a black box on a dark gray background was presented for 3 s; it was either repeated from the cue period (match) or represented a novel item (non-match). Participants were prompted to make a button press response indicating their match/non-match discrimination.

The load and affect conditions for the cue period, the type and affect of the distractor, and the match or non-match status of the decision stimulus were equally balanced across MRI sessions (Fig. 1). For trials to be considered correct for subsequent behavioral and fMRI analyses, participants needed to both accurately identify the type of distractor and make a valid match/non-match discrimination at probe (see also Supplementary Methods). All of the following statistical tests were two-sided.

### Behavioral analysis

Behavioral data from the fMRI working memory task were summarized in MATLAB (https://www.mathworks.com/) and analyzed in R (https://www.r-project.org/). Performance was assessed by metrics including the mnemonic discriminability (d′), also referred to as the sensitivity index, and the response bias (criterion c) taken from signal detection theory [32]. Both measures were calculated using the hit rate, defined as the proportion of trials in which subjects correctly recognized a repeated stimulus presented at decision, and the false alarm rate, defined as the proportion of trials in which subjects falsely recalled a novel stimulus presented at decision. These rates were normalized using the inverse of the cumulative standardized normal distribution (qnorm function) prior to the calculation of the performance metrics. The sensitivity index was calculated by subtracting the normalized false alarm rate from the normalized hit rate. Response bias was calculated as the negative average of the normalized hit rate and the normalized false alarm rate (–½(normalized hit rate + normalized false alarm rate)). The behavioral data were analyzed with maximum likelihood-based linear mixed effects models (“lmer” function) predicting the behavioral metric with the mean-centered covariates of interest—i.e., including a task factor and/or the study drug intervention, as well as their interactions [33]. The primary covariate of interest was PTSD severity, but both baseline working memory span and number of mild traumatic brain injury events were also assessed to determine the selective influence of PTSD symptomatology.

### MRI data acquisition and univariate analysis

MRI scanning was performed with a Siemens 3 Tesla Siemens TIM/Trio scanner at the Henry H. Wheeler, Jr. Brain Imaging Center at the University of California, Berkeley. Image preprocessing and data cleaning were conducted using standard procedures (see Supplementary Methods). Event-related univariate fMRI analysis was performed using variable duration boxcars with onsets corresponding to the beginning of the cue phase, the onset of the distractor, and the decision phase; durations were 2.7, 2.5, and 3 s, respectively. These boxcars were then convolved with the canonical hemodynamic response function. Regressors of interest specified whether the trial was correct or incorrect for each task phase and facial emotion for stimulus emotion models (e.g., correct fearful cue, correct fearful distractor, and correct fearful decision stimulus) to maximize statistical power for each model of interest. These regressors, as well as regressors of no interest, were estimated after concatenating all scan runs (function “spm_fmri_concatenate.m”). Second-level random effects testing was performed over the first-level univariate parameter estimate contrast images, along with the mean-centered behavioral covariate of interest and the study drug intervention. For detailed descriptions of the analysis methods, see Supplementary Methods.

### Generalized psychophysiological interactions analysis

Brain regions for functional connectivity analyses (“seeds”) were identified as areas that demonstrated significant interactions with PTSD severity. Specifically, spherical regions centered on the MNI coordinates of the statistical maxima of significant clusters were created with radius 5.3 mm (two voxels) using the WFU PickAtlas SPM toolbox (https://www.nitrc.org/projects/wfu_pickatlas/). These seed regions were then refined via intersection with the second-level mask to ensure that only statistically significant voxels were included. Psychophysiological interactions (PPI) analysis was performed with these functional connectivity seeds using the Generalized PPI Toolbox (https://www.nitrc.org/projects/gppi) [34]. First-level models for the PPI analysis expanded the general linear models employed for univariate fMRI analysis to include additional regressors representing the first eigenvariate of the timecourse of the seed region, as well as the interaction between the seed timecourse and the task regressors for each model. Group results based on first-level PPI regressors were assessed with second-level random effects testing that included the mean-centered behavioral covariate of interest and the study drug intervention. Follow-up PPI results were restricted to the same task phase as the univariate analysis from which the seeds were derived (e.g., the decision phase).

## Results

### Behavioral analysis

**Fig. 2:**
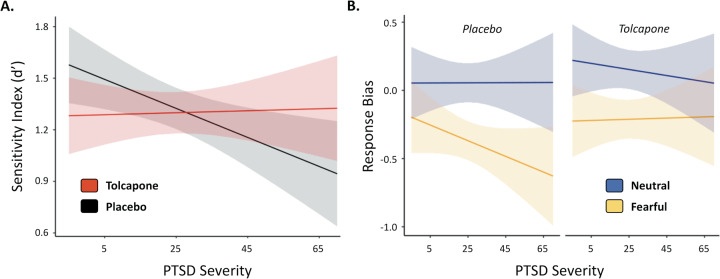
Interactions between PTSD severity, stimulus emotion, and drug condition predict working memory performance. **A** PTSD severity and drug condition interact (*p* = 0.0184), such that participants with higher CAPS-5 scores demonstrate a greater sensitivity index on tolcapone, while subjects with lower CAPS-5 scores perform better on placebo. **B** PTSD severity interacts with stimulus emotion and drug session to predict response bias (*p* = 0.0309), such that participants with more severe PTSD are more biased in responding to fearful as compared to neutral stimuli. This effect is ameliorated on tolcapone. Errors represent 95% confidence intervals.

To evaluate the effects of PTSD severity and the influence of tolcapone on working memory, an affective working memory task was used to assess military veterans with a range of PTSD symptomatology (CAPS-5 scores 0–68, mean 25.4, where scores of 31–33 or higher suggest the need for PTSD treatment—https://www.va.ptsd.gov). Independent linear mixed models evaluated predictors of mnemonic discriminability (i.e., the sensitivity index, or d′) and response bias, respectively, as we did not have a priori hypotheses about which behavioral metric would mediate our behavioral effects. To evaluate our hypothesis of an inverted-U dose-response for tolcapone and PTSD, we examined a model predicting d′ from mean-centered PTSD severity, drug intervention (tolcapone versus placebo), and their interaction. PTSD severity (F(1,30) = 2.23, *p* = 0.146) and drug intervention (F(1,30) = 0.131, *p* = 0.720) were not significant predictors alone, but their interaction was significant (F(1,30) = 6.213, *p* = 0.0184, adj. *R*^2^ = 0.1440; Fig. 2A). To better understand the direction of this interaction, we compared tolcapone versus placebo at different estimated marginal means for CAPS-5 scores (one standard deviation below average, average, and both one and two standard deviations above average) to estimate the effect of drug across a range of PTSD severities. At one standard deviation below average PTSD severity, performance approached significant worsening on tolcapone versus placebo (t(32.1) = −1.956, *p* = 0.0592, *d* = 0.6905), while at two standard deviations above average PTSD severity, performance approached significant improvement on tolcapone versus placebo (t(32.1) = 2.002, *p* = 0.0538, *d* = 0.7067). There were no significant differences at mean PTSD severity or one standard deviation above average. In keeping with an inverted-U shaped relationship between tolcapone response and PTSD severity, participants with low PTSD severity, and presumptively normal cortical dopamine tone, performed worse on tolcapone, while those with more severe PTSD, and presumptively reduced cortical dopamine tone, showed improvements. We also assessed the effect of PTSD severity and tolcapone on response bias, rather than the sensitivity index, independent of other task factors. In a model including mean-centered PTSD severity, drug, and their interaction, there was no effect of PTSD, but the drug intervention was a significant predictor (F(1, 30) = 5.649, *p* = 0.0241, adj. *R*^2^ = 0.1304). A post hoc test revealed that subjects responded significantly more conservatively on tolcapone compared to placebo (t(32.1) = 2.296, *p* = 0.0283, *d* = 0.8105).

After establishing that the tolcapone intervention improved working memory performance in subjects with greater PTSD symptomatology, we aimed to assess whether tolcapone would improve performance for more severe PTSD subjects for emotionally arousing stimuli. Specifically, we examined the influence of stimulus emotion (fearful vs. neutral affect) on the sensitivity index and response bias performance metrics. We also performed exploratory analyses on memory load (high vs. low load) and distractor type (face vs. place) task factors. While the task factors of memory load and distractor type did not interact significantly with the drug intervention and PTSD severity, stimulus emotion did. For the sensitivity index, a model using stimulus emotion, drug intervention, mean-centered PTSD severity, and the associated interactions as predictors identified a significant main effect of stimulus emotion and an interaction between stimulus emotion and PTSD severity, but no effect of drug (see Supplementary Results). However, for response bias, stimulus emotion (F(1, 30.0) = 69.857, *p* < 0.0001, adj. *R*^2^ = 0.6896), drug intervention (F(1, 30.0) = 5.213, *p* = 0.0297, adj. *R*^2^ = 0.1196), and the interaction between PTSD severity, stimulus emotion, and drug intervention (F(1, 30.0) = 5.132, *p* = 0.0309, adj. *R*^2^ = 0.1176) were significant predictors (Fig. 2B). Post hoc paired *t*-tests showed that neutral cue stimuli resulted in more conservative responding than fearful cue stimuli (t(32.1) = 8.075, *p* < 0.0001, *d* = 2.8505) and that tolcapone also resulted in more conservative responding than placebo (t(32.1) = 2.206, *p* = 0.0347, *d* = 0.7787). However, the 3-way interaction appeared to be driven by a more liberal response bias to fearful stimuli in subjects with greater PTSD severity, a bias that lessened on tolcapone versus placebo (Fig. 2B and Supplementary Results).

### fMRI analysis

To assess the neural mechanisms for these behavioral interactions with PTSD severity, we performed separate univariate fMRI analyses. Given the findings for the sensitivity index, which was predicted by the interaction between drug condition and PTSD severity, we analyzed this same interaction (i.e., between drug condition and CAPS-5 score) in the imaging data. For response bias, which was predicted by the interaction between drug condition, stimulus emotion, and PTSD severity, we similarly evaluated these factors during the cue and decision phases. Specifically, we computed the interaction term for a two factor repeated measures ANOVA contrasting neutral with fearful stimuli and tolcapone with placebo, then regressed the result against CAPS-5 score. Because prior studies have not converged on a single memory phase that is affected in PTSD [14, 15, 35], we did not have strong a priori hypotheses about the task phase in which these changes would occur. Consequently, we examined both the cue and decision phases.

**Fig. 3:**
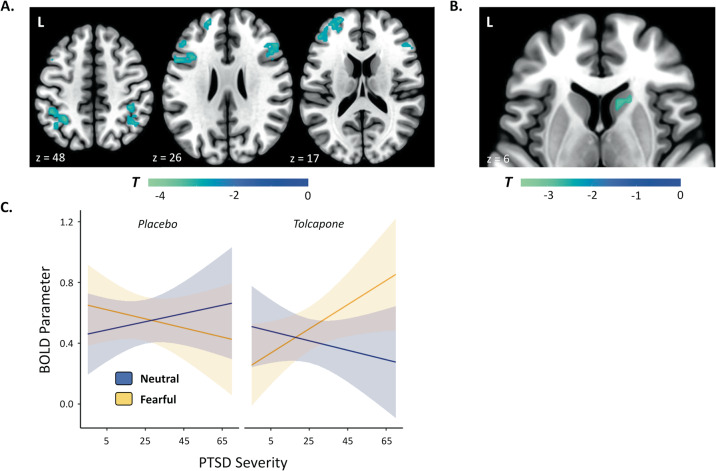
The interaction between PTSD severity, stimulus emotion, and drug condition predicts BOLD responses. **A** Brain regions demonstrating an interaction between drug condition, stimulus emotion, and PTSD severity at decision. All voxels shown exceed a threshold of *p* < 0.05, corrected. **B** An area within the head of the caudate also shows an interaction between drug condition, stimulus emotion, and PTSD severity at decision. All displayed voxels exceed a threshold of *p* < 0.05, small-volume corrected. **C** PTSD severity interacts with stimulus emotion and drug session to predict BOLD parameters for the brain regions presented in (**A**) and (**B**) (*p* < 0.0001). Post hoc tests revealed that for PTSD subjects one (*p* = 0.0003) and two standard deviations (*p* = 0.0001) above average PTSD severity, significantly stronger BOLD responding for fearful above neutral stimuli was seen on tolcapone. For subjects one standard deviation below average PTSD severity, BOLD responding declined for fearful stimuli on tolcapone compared to placebo (*p* = 0.0498). Note that the 3-way interaction from the univariate analysis is not an independent statistical test, but was undertaken to enable post hoc tests and to visualize the underlying pattern of results. Errors represent 95% confidence intervals.

No significant whole-brain neuroimaging findings were seen for the interaction between drug condition and PTSD severity, whether analyses were conducted in the cue or decision phase. However, for the interaction between drug condition, stimulus emotion, and PTSD severity, significant clusters were identified in bilateral frontoparietal areas during the decision phase of the task (Fig. 3A). The specific set of brain regions that emerged from this analysis included clusters in the left frontal pole, the bilateral inferior and middle frontal gyri, and the bilateral intraparietal sulcus. Moreover, an accompanying region of interest analysis in the striatum identified a significant 18 voxel right caudate cluster (Fig. 3B). See Supplementary Figure 3 for parameter estimates derived from the 3-way interaction between drug condition, stimulus emotion, and PTSD severity (split into high and low CAPS-5 subjects for visualization purposes) for all clusters and Supplementary Table 2 for MNI coordinates and cluster information.

To understand the nature of this effect more clearly, we averaged the BOLD parameters across the network of significant brain regions, including both frontoparietal regions and caudate, and used the result to predict the fMRI responses in a linear mixed model consisting of centered PTSD severity, stimulus emotion, the drug intervention, and their interactions as regressors. As expected, this analysis confirmed the 3-way interaction between stimulus emotion, PTSD severity, and the drug intervention (F(1, 60.0) = 24.118, *p* < 0.0001, adj. *R*^2^ = 0.2748; see Fig. 3C). Post hoc analyses compared the paired effects of the drug intervention and stimulus emotion across PTSD severities using estimated marginal means, where *p* values were corrected for the family of 4 estimates assessed at one standard deviation below average, average, and both one and two standard deviations above average PTSD severity, respectively. The comparison assessing BOLD responding between neutral and fearful stimuli on tolcapone showed that activity was significantly greater for fearful above neutral stimuli for CAPS-5 scores one standard deviation above (t(64.3) = 4.297, *p* = 0.0003, *d* = 1.0717) and two standard deviations above average severity (t(64.3) = 4.697, *p* = 0.0001, *d* = 1.1715). In contrast, for one standard deviation below average PTSD severity, there was a significant effect of lower BOLD responding for fearful stimuli on tolcapone compared to placebo (t(50.1) = 2.659, *p* = 0.0498, *d* = 0.7513). To summarize, tolcapone resulted in increased activations to fearful stimuli above neutral stimuli for higher severity PTSD subjects, while lower severity PTSD subjects exhibited reductions in fMRI activity for fearful stimuli compared to placebo in these regions. Next, to assess whether there was a relationship between these frontoparietal and caudate activations and the behavioral measures previously described (d′ and response bias), we correlated the average frontoparietal and caudate BOLD parameters for the interaction of drug condition, stimulus emotion, and PTSD severity with the like interaction for the sensitivity index and response bias measures. Across all subjects, we found a significant correlation with response bias (*r* = 0.3711, *p* = 0.0435), while the correlation with sensitivity index was not significant (*r* = −0.2995, *p* = 0.1079), thereby providing converging evidence for the connection between behavioral responding, as indexed by the response bias, and frontoparietal recruitment on tolcapone.

**Fig. 4:**
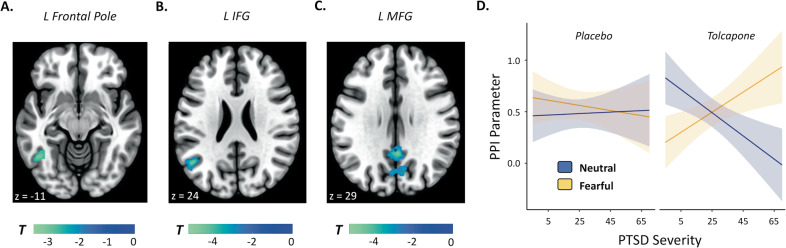
Brain regions whose connectivity with the listed seed region demonstrates an interaction between drug condition, stimulus emotion, and PTSD severity. Respective seed regions include **A** the left frontal pole, **B** the left inferior frontal gyrus, and **C** the left middle frontal gyrus. All voxels shown exceed a threshold of *p* < 0.05, corrected. **D** PTSD severity interacts with stimulus emotion and drug session to predict PPI parameters for the brain regions presented in (**A**), (**B**), and (**C**) (*p* < 0.0001). Similar to the univariate analysis, post hoc tests revealed that for PTSD subjects one (*p* = 0.0001) and two standard deviations (*p* < 0.0001) above average PTSD severity, significantly greater PPI coupling for fearful above neutral stimuli was seen on tolcapone. For subjects one standard deviation below average PTSD severity, PPI network coupling declined for fearful stimuli on tolcapone compared to placebo (*p* = 0.0489), and increased for neutral above fearful stimuli on tolcapone (*p* = 0.0001). Note that the 3-way interaction from the PPI analysis is not an independent statistical test, but was undertaken to perform post hoc tests and to visualize the underlying pattern of results. Errors represent 95% confidence intervals.

To assess how these prefrontal responses might be communicated to the rest of the brain, we next performed an exploratory analysis evaluating each of the PFC clusters from the univariate analysis—the frontal pole, the middle frontal gyrus, and the inferior frontal gyrus—to determine whether their functional connectivity differed for memory judgments during decision as a function of drug condition, stimulus emotion, and PTSD severity. Significant connectivity changes were evident. The left frontal pole cluster demonstrated a significant 3-way interaction with a left inferior temporal region (Fig. 4A); the left inferior frontal gyrus demonstrated a 3-way interaction with a left angular and middle temporal gyrus cluster (Fig. 4B); and the left middle frontal gyrus exhibited 3-way interactions with the bilateral posterior cingulate cortex region and cuneus, both of which extended into the precuneus (Fig. 4C). See Supplementary Figure 4 for parameter estimates and Supplementary Table 2 for MNI coordinates and cluster information.

The nature of these effects in areas classically associated with the default mode network was consistent with that of the univariate findings. To assess the network of PPI connections, we averaged the PPI parameters across the network of significant brain regions including left temporal gyrus, left angular gyrus, right posterior cingulate cortex, and left cuneus to predict the PPI responses in a linear mixed model using centered PTSD severity, stimulus emotion, the drug intervention, and their interactions as regressors. Similar to the univariate fMRI post hoc analysis, we replicated the 3-way interaction (F(1, 60.0 = 30.701, *p* < 0.0001, adj. *R*^2^ = 0.3275; see Fig. 4D) and also found an interaction between PTSD severity and stimulus emotion (see Supplementary Results). Post hoc tests using estimated marginal means across the previous family of 4 PTSD severity levels replicated the three post hoc findings from the fMRI analysis for the PPI network coupling analysis, as well as additional PPI connectivity effects. Specifically, for PTSD subjects one (t(64.3) = 4.703, *p* = 0.0001, *d* = 1.1730) and two (t(64.3) = 5.910, *p* < 0.0001, *d* = 1.4740) standard deviations above average PTSD severity, tolcapone led to PPI coupling that was greater for fearful than neutral stimuli. In contrast, for subjects one standard deviation below average PTSD severity, PPI coupling increased for neutral above fearful stimuli on tolcapone (t(64.3) = 4.604, *p* = 0.0001, *d* = 1.1483)—i.e., opposite the pattern seen in higher severity PTSD patients. Neither the sensitivity index nor response bias correlated significantly with these PPI changes (both *p* values >0.45 (n.s.)).

## Discussion

PTSD and anxiety disorders are thought to impact cognition, and cognitive impairments associated with such disorders are capable of independently limiting daily function. To directly assess these cognitive deficits, this study evaluated the ability of a novel dopaminergic intervention to improve working memory and affective functioning in military veterans with a range of PTSD severity. In addition to confirming that higher PTSD severity correlates with reduced performance on an emotional working memory task [2], our results demonstrate that tolcapone, a drug that enhances cortical dopamine tone via inhibition of the COMT enzyme [17, 20], partially remediates this working memory dysfunction in subjects with more severe PTSD symptoms. In addition to boosting working memory performance as assessed by the sensitivity index, this dopaminergic intervention decreased liberally-biased responding to fearful cues for more severe PTSD subjects, reducing a potential source of emotional impairment. Notably, fMRI demonstrated that the improvements in response bias for more severe PTSD subjects, particularly at the time of decision, were linked to activity within frontoparietal (and striatal) regions commonly implicated in working memory, and accompanied by changes in connectivity between frontal regions and areas previously linked to the default mode network.

These behavioral findings build upon previous work demonstrating that cognitive and affective deficits are often comorbid [2, 36]. The impairments in processing of fearful stimuli found here, for example, are consistent with a recent meta-analysis demonstrating that reduced working memory performance in task conditions including negative affect is particularly prevalent in populations suffering from mental health disorders [37]. Relatedly, although tolcapone improved working memory performance in subjects with more severe PTSD symptoms, it modestly reduced performance in subjects without clinical PTSD, supporting past work that measures of working memory typically exhibit an inverted U-shaped dose response to dopamine, in which too little and too much dopamine both impair performance [3, 4, 38]. A decrement in performance was less evident in the response bias: liberal responding to fearful stimuli declined on tolcapone in subjects with higher but not lower CAPS-5 scores, though it is possible that the trauma-exposed controls studied here might demonstrate more modest declines than controls who did not have such exposures.

More mechanistically, our neuroimaging results identified the strongest neural changes during the decision (i.e., retrieval) phase of our task. Depending on the study, alterations in working memory processes have previously been found during the cue [15, 35], maintenance/distractor [36, 39, 40], and decision [14] phases. Although the reasons for this variability are unclear, previous work suggests that subjects with PTSD may overgeneralize from negative to more neutral stimuli or features [41]. In addition, it has been suggested that working memory in PTSD subjects may be especially impaired with negative or trauma-related material [36, 40], and that PTSD subjects may have particularly limited executive resources to support working memory [42, 43]. In the current paradigm, in which cue and decision face emotion were always congruent, failure to engage control mechanisms to reduce attention to emotion during the decision phase may therefore reduce the accuracy of a match/non-match decision that should be based not on affect, but on other stimulus features. Consistent with this idea, our neuroimaging results show that tolcapone upregulates the recruitment of frontoparietal regions during the decision phase, suggesting that engagement of these presumptive control areas may represent a source of improved working memory and emotional performance for more severe PTSD subjects. Specifically, 3-way interactions between PTSD severity, stimulus emotion, and the drug intervention identified areas overlapping the canonical frontoparietal control network (FPCN) [44, 45], including clusters in the bilateral inferior and middle frontal gyri, bilateral intraparietal sulcus, and left frontal pole, that tracked these experimental factors.

Using a small volume correction, we also identified a corresponding interaction in the head of the right caudate nucleus, in keeping with the known importance of frontal-striatal-thalamic loops in higher cognitive function [46]. We speculate that this caudate activation may be due to its well-characterized reciprocal connections with the PFC [47], rather than direct dopaminergic modulation via COMT inhibition. Most extant research has shown that COMT clearance is a comparatively minor pathway for dopamine metabolism from the striatum [18, 21, 23], although the direct explanation cannot be entirely excluded due to prior research in rodents demonstrating an increase in striatal dopamine metabolites following tolcapone administration [48].

Notably, for subjects with greater CAPS-5 scores, these frontoparietal brain (and striatal) regions resulted in significantly greater activation for fearful above neutral stimuli on tolcapone, arguing that enhancement of cortical dopamine tone permitted recruitment of greater cognitive control resources for these stimuli in these individuals, leading to reduced response bias. Prior psychiatric theories have suggested that PTSD is associated with “hypofrontality,” in which reduced prefrontal cortical responding, potentially due to synaptic loss from chronic stress [49], leads to behavioral impairments [50]. Our findings suggest that augmenting activity in specific frontoparietal regions, here using a dopamine manipulation, may improve cognitive control. Moreover, the finding that these patterns of frontoparietal and striatal recruitment correlate with the changes in response bias found in the behavioral analyses provides converging support that tolcapone may improve emotional regulation in PTSD subjects. This effect may be to reduce liberally-biased responding, potentially a manifestation of overgeneralized fear responses [41, 51], through its effects on frontoparietal networks associated with cognitive control and schematic processing [52].

In addition, greater engagement of these frontoparietal areas was accompanied by changes in connectivity between prefrontal regions and areas that have been linked to the default mode network (DMN). Our PPI functional connectivity analysis found that the left frontal pole showed an interaction with the left inferior temporal cortex, the left inferior frontal gyrus (IFG) with the left angular and middle temporal gyri, and the left middle frontal gyrus (MFG) with the posterior cingulate, cuneus, and precuneus. The pattern of functional connectivity effects was consistent with that from the univariate analysis: on tolcapone, subjects with higher CAPS-5 scores exhibited greater PPI coupling for fearful above neutral stimuli. While our study was not designed to evaluate other behavioral functions of these posterior cortical regions, previous work has shown that they may play a role in internally-oriented cognition [53, 54], and the angular gyrus itself may be important for the integration of information in working memory [55]. We speculate that to make judgments as to whether the decision face has been previously encountered, subjects require a high-fidelity neural representation of the stimulus (or stimuli) for a proper comparison. In long-term memory studies, increased functional connectivity between similar prefrontal and posterior cortical areas is important for memory retrieval judgments [56–58], and by analogy, better access to representations held in the angular gyrus may be important for the current task [59]. Alternatively, the bilateral posterior cingulate, precuneus, and cuneus are associated with abnormal responding in PTSD subjects, with the posterior cingulate and precuneus showing strong responses to intrusive trauma imagery [11]. Consistent with prior theories, it is thus possible that tolcapone helps more severe PTSD subjects exert greater control over, and thereby limit the influence of, traumatic imagery, reducing liberal bias to fearful cues.

Although this paper is focused on the effects of dopamine on working memory and emotion regulation in higher severity PTSD subjects, we note that there were significant findings for the lower severity PTSD subjects as well, who effectively served as trauma-exposed controls in this study. Although the low CAPS-5 subjects did not show a clear behavioral correlate of stimulus emotion, they did demonstrate reduced working memory performance on tolcapone, consistent with the consequences of excessive dopaminergic stimulation [3]. Such “overdose” effects might disrupt the balance between working memory maintenance and gating processes [60] that depend on coordinated reciprocal interactions between the PFC and the striatum [47], potentially complemented by interactions between frontoparietal regions and the DMN [61]. In PPI analyses in lower severity subjects, tolcapone compared to placebo led to reduced BOLD responding and PPI coupling for fearful stimuli. Moreover, these subjects exhibited greater PPI coupling for neutral above fearful stimuli on tolcapone, in contrast with the PTSD subjects. Excessive dopamine responding is associated with acute stress and tracks with the intensity of perceived psychosocial stress, with effects in the PFC linked to increased threat monitoring [62]. It is therefore possible that elevated dopamine tone due to tolcapone mimicked a mild impairment in emotional working memory processing due to stress, though more work would be necessary to determine whether dopamine tone alone could induce this effect.

Although these findings suggest that enhancing cortical dopamine tone in subjects with more severe PTSD symptomatology can potentially enhance working memory function, a number of caveats should be noted. While functional neuroimaging studies are important for understanding neural mechanisms, their demands on subjects and their expense limit the overall number of participants. Further studies in larger cohorts will be important to confirm the behavioral and neuroimaging findings identified here. In addition, while we had predicted that tolcapone-induced changes in the sensitivity index would also track activity in frontoparietal regions, we were unable to find neuroimaging correlates for this important behavioral finding. Although unable to definitively account for this null result, we speculate on possible explanations. The change in information processing associated with improved working memory in PTSD subjects could be instantiated by activity patterns that reorganize without causing changes in univariate BOLD activity [63]. Unfortunately, the task design in our study was not optimized for analytic approaches such as multi-voxel pattern analysis, so future research will have to be undertaken to assess this possibility. For similar reasons, we would not have detected activity-silent reorganization of synaptic weights [64]. Last, it is possible that we were unable to find fMRI correlates for this effect because we lacked either the spatial resolution to detect small but meaningful BOLD activations, or the statistical power to find small effects. In addition to studies with greater statistical power, future studies can employ designs that allow for multi-voxel pattern analysis methods with high-resolution brain data to begin to address these outstanding questions.

Future research might also investigate whether additional factors impact the relationship between PTSD, working memory gating, and dopamine. Our study did find modest but inconclusive evidence to identify concentration problems as a mediator of the effect of PTSD on working memory impairment (see Supplementary Results). In addition, our focus on combat PTSD in military veterans led to a significant gender imbalance (25 men, 5 women), and thus we were unable to further explore known gender-specific factors, such as estradiol levels, that modulate dopaminergic influences on working memory function [65]. Furthermore, previous research has suggested that the cognitive effects of tolcapone depend on a functional COMT polymorphism (rs4680), with individuals who are homozygotic for the more active Val-encoded enzyme tending to exhibit working memory and cognitive improvements, while homozygotic Met allele carriers tend to exhibit worse performance [20, 26, 38]. Our study was unable to find a relationship between PTSD, COMT genotype, and working memory (see Supplementary Results), although our analyses of genotype subgroups were underpowered. Future research should attempt to assess the role of COMT genotype on PTSD and dopamine status with a larger sample size. Last, changes in performance on cognitive testing in the laboratory, in which the environment is relatively controlled, do not always translate to more naturalistic settings. Longer-term, real-world studies—including work in individuals with PTSD due to other etiologies—would be a necessary next step to determine the efficacy of tolcapone in remediating cognitive deficits in the setting of PTSD.

Despite these caveats, these findings emphasize the comorbidity of both affective and cognitive impairments, and they point to the potential for focused treatment of cognitive deficits in PTSD subjects. More directly, based on extensive past work demonstrating the importance of cortical dopamine to working memory function, here we show proof of principle evidence that enhancing cortical dopamine tone via COMT inhibition can lead to improvements in cognitive control in subjects with more severe PTSD symptoms. Given the aging of the veteran population [66] and their comparatively higher rates of mental health disorders including PTSD (a known risk factor for dementia [67]), future studies might investigate whether tolcapone or similar dopaminergic interventions could mitigate the cognitive dysfunction associated with both PTSD and aging. Whether or not such therapies are ultimately related to dopamine, understanding how this and other treatments might remediate working memory impairments in such individuals in more naturalistic settings remains an important goal for future work.

## Supplementary information

Supplemental Material
