# Supplementary material for: Working memory, cortical dopamine tone, and frontoparietal brain recruitment in post-traumatic stress disorder: a randomized controlled trial
Source: Transl Psychiatry. 2021 Jul 12;11:389. doi: 10.1038/s41398-021-01512-6 (PMC8275779; doi:10.1038/s41398-021-01512-6)
Supplement: Supplementary file 1 — Supplemental Material [file 41398_2021_1512_MOESM1_ESM.docx]

**Supplementary Methods**

*Inclusion Criteria.* Subjects were enrolled as part of a larger effort to understand the effects of PTSD and concussion (mild traumatic brain injury) on cognitive function. To take part, participants were required to meet the following inclusion criteria:

1. between the ages of 18 and 50 years old
2. able to read English at a 6^th^ grade level as determined by the Wechsler Test of Adult Reading (WTAR)
3. able to provide written informed consent
4. a history of mild traumatic brain injury (Glasgow Coma Scale (GCS) score between 13 and 15, loss of consciousness < 30 min, and/or post-traumatic amnesia < 24 hours from onset) as confirmed by structured clinical history greater than 6 months prior to participation
5. normal or corrected-to-normal visual acuity
6. stable doses of all medications (2 weeks or greater)

*Exclusion Criteria.* Additionally, participants were excluded if they met any of the following criteria:

1. contraindications to MRI (e.g., unremovable ferromagnetic metals, claustrophobia)
2. inability to complete basic fMRI requirements (e.g., making button presses, minimizing movement < 5 mm)
3. contraindications to tolcapone use prior to receipt of active study medication, including any liver function test (Aspartate Aminotransferase Test, Alanine Aminotransferase Test, Total Bilirubin, Direct Bilirubin, Alkaline Phosphatase) elevated more than 2 times above the upper bound of the normal range, previous adverse reaction to tolcapone, pregnancy, or significant liver impairment including but not limited to chronic hepatitis, cirrhosis, hepatocellular carcinoma, parasitic liver infection, autoimmune hepatitis, primary biliary cirrhosis, hemochromatosis, or any other liver condition obtained from patient history and/or available medical records, as identified by the reviewing clinician (A.S.K.).
4. any use within the previous 30 days of pharmacological agents with dopaminergic actions prior to receipt of active study medication, including but not limited to tolcapone, levodopa/carbidopa, entacapone, amantadine, bromocriptine, pergolide, pramipexole, ropinirole, selegiline, isocarboxazid, phenelzine, tranylcypromine, clozapine, olanzapine, quetiapine, risperidone, ziprasidone, aripiprazole, fluphenazine, haloperidol, perphenazine, pimozide, thiothixene, trifluoperazine, loxapine, molindone, chlorpromazine, mesoridazine, thioridazine, promethazine, dextroamphetamine, dexmethylphenidate, dextroamphetamine, methylphenidate, cocaine, or methamphetamine
5. use of an investigational drug within 30 days of screening visit
6. alcohol dependence or alcohol abuse within the last 30 days by DSM-IV-TR criteria
7. active substance dependence or substance abuse by DSM-IV-TR criteria within the previous 30 days, including marijuana, opiates, cocaine, amphetamines, and hallucinogens
8. a previous diagnosis of substance abuse or a positive urine drug screen for illicit substances during the study (e.g., amphetamine, cocaine, and other drugs)
9. a past diagnosis of schizophrenia, attention-deficit hyperactivity disorder, and/or other psychiatric diagnosis except depression or PTSD
10. a history of psychiatric hospitalization (last 1 year), suicide attempt (last 5 years), or current suicidal ideation
11. a history of major brain surgery or penetrating brain injury (e.g. violating brain parenchyma)
12. the presence of seizures persisting more than 4 weeks beyond a documented inciting event, or requiring active pharmacological treatment
13. a history of brain tumor, stroke, demyelinating disease, encephalitis, or aneurysm rupture
14. a diagnosis of Alzheimer’s disease or other primary neurodegenerative disorder
15. severe low blood pressure or uncontrolled high blood pressure
16. any clinically severe medical illness requiring treatment

The participants did not have neuroanatomical abnormalities on neuroimaging, as reviewed by a neurologist (A.S.K.), had normal or corrected-to-normal vision, and were right-handed. Data collection for this study was performed between November 2015 and May 2018, and ended when the study was adequately powered (see next section).

*Power Analysis.* As our study primarily represents a within-group, repeated-measures design with comparisons between more and less severe PTSD subjects, we calculated that for a 2-way ANOVA with 2 groups and repeated measurements that are moderately correlated (r = 0.6), 28 subjects would provide 80% power to detect a difference of moderate effect size (0.25) at a significance level of p < 0.05.

*Screening Measures.* Demographic information and clinical measures were collected at a screening visit prior to drug administration and MRI data collection. PTSD severity at the time of the research study was measured using the CAPS-5, a time-intensive, and potentially emotionally charged, instrument for which the typical evaluation lasted 45 minutes. For these reasons, we performed it at the screening visit, rather than on testing days, and did not repeat it after the initial evaluation. On average, the time between the screening visit and the first scan session was 20.6 days (SD = 18.3 days) followed by an average of 18.5 days (SD = 17.1 days) until the second scan was completed. When the groups were split by whether they received tolcapone or placebo at the first scan session, these statistics related to study duration were nearly identical (data not shown). We did not expect there to be wide fluctuations in the PTSD symptoms in our participants over time, consistent with the good test-retest reliability of the CAPS over short time periods ^1^ and the chronicity of our subjects’ symptomatology. For a distribution of PTSD severity measured by the CAPS-5, see **Figure S2**. 18 participants in our study had been diagnosed with a lifetime history of PTSD, independent of CAPS-5 score.

The Center for Epidemiologic Studies Depression Scale – Revised (CESD-R) ^2^ was used to assess major depressive disorder symptomology. Scores for 16 subjects indicated no clinically significant depression, while scores for 14 subjects suggested the presence of clinically subthreshold depressive symptoms. Attention difficulties in our participants were measured with the Barratt Impulsiveness Scale (BIS) ^3^ attention subscale. 4 subjects had scores indicating rare concentration difficulties, 11 subjects had scores indicating occasional concentration problems, 14 subjects had scores specifying frequent attention problems, and 1 patient had a score indicating concentration difficulties almost always. Premorbid intelligence was assessed with the WTAR ^4^. See **Table S1** for more information.

*COMT Genotyping.* Participants were evaluated for their COMT genotype profile by DNA extraction and SNP analysis performed at the UCSF Institute for Human Genetics on salivary samples (salimetrics.com) collected at the time of the screening visit. Gentra Puregene reagents and protocols were used to extract DNA, which was quantified with the Pico Green method (Molecular Probes/Invitrogen). Genotyping was assessed on the catechol-O-methyltransferase (COMT; rs4680) polymorphism using TaqMan® technology (Applied Biosystems) and was available for 22 out of 30 participants who completed the study.

*Data Collection.* T1-weighted anatomical images were collected with an MP-RAGE protocol (160 slices, TR = 2300 ms, TE = 2.98 ms, FoV = 256 mm, matrix size = 256 x 256, voxel size = 1 mm^3^). Functional MRI (fMRI) data were acquired using a gradient echoplanar imaging protocol (24 slices in ascending interleaved order, TR = 1840 ms, TE = 33 ms, FoV = 225 mm, matrix size = 96 x 96, voxel size = 2.3 x 2.3 x 3.5 mm). The working memory task was displayed using a projector (Avotec SV-6011, [http://www.avotecinc.com](http://www.avotecinc.com/), Stuart, Florida) and a translucent screen placed within the scanner bore behind the head coil. Participants viewed the screen via a head coil-mounted mirror and made task responses with an MRI-safe fiber optic response pad (Inline Model HH-1 × 4-L, [http://www.crsltd.com](http://www.crsltd.com/), Rochester, Kent, UK). Participants underwent fMRI scanning while completing runs from this task in pseudorandom order. The task consisted of six 8 minute, 15 second runs of 23 trials and one run of 22 trials. Each trial was 21.2 s in duration on average, with a jittered inter-trial interval of 1-7 s (see below). In addition. a resting state run was collected, though it was not further assessed for this study. Stimuli were presented and responses recorded for the working memory task using the E-Prime experimental design software package (Psychology Software Tools, Inc., Pittsburgh, PA). On the 4-button response box, the left-most button was used to select the presented left option (“Face” for distractor trials, “Yes” for decision trials) and the rightward adjacent button used to select the right option (“Place” for distractor trials, “No” for decision trials; see below).

*Task.* At the beginning of each trial, three faces comprising the cue stimuli were presented for a total of 2.7 s on a black box within a grey background, followed by the initial delay (see below). A distractor stimulus consisting of either a face or a place image was then displayed within a dark gray box on a black background for 2.5 s, followed by the second delay. Lastly, an image of a probe (decision) face was presented for 3 s within a black box on a dark grey background, followed by the inter-trial interval. The delays immediately preceding and following the distractor stimulus were jittered in coordinated fashion, lasting 1.5 (5.5), 3.5 (3.5), or 5.5 (1.5) seconds before (after) the distractor, for a total of 7 s per trial. The trial cue, jittered delays, and inter-trial interval all included a central fixation cross on a white background; the fixation cross preceding trial onset was gray rather than black in order to provide an additional cue to the beginning of the trial. None of the stimulus images were repeated, except when the decision stimulus was a match for one of the cue stimuli on a given trial.

Both cue and decision face stimuli were randomly generated using FaceGen’s built-in random face generation option (<https://facegen.com/>). They were allowed to freely vary by gender, age, and race. None of the stimuli had facial blemishes or hair in order to reduce the number of distinguishing non-facial features available at decision. The neutral distractor scenes were chosen to represent a random sampling of outdoor landscapes (e.g., mountains, plains, and rivers), while the arousing scenes were selected to exemplify military-related themes without faces or readily visible human forms (e.g., military equipment and scenery from Operation Enduring Freedom and Operation Iraqi Freedom). The distractor faces were either fearful or neutral in affect. These stimuli were taken from a validated German sample of emotional expressions, were permitted to vary in gender and age, but race (Caucasian) was fixed. (To allow us to include variations in race, and to account for the large number of face stimuli required, these distractor faces were not used in the cue phase.) Because cue faces were computer-generated, and distractor faces were not, no trial-wise matching was performed between cue and distractor stimuli. For the two study sessions, two unique sets of stimuli were created and randomly paired with one of the two MRI sessions across participants, except for the final four subjects, for whom the pairing was set to balance the frequency with which each stimulus set was shown first and second. All task conditions were counterbalanced to ensure that each unique combination of cue, distractor, and decision conditions was present in each scanner run.

*MRI Preprocessing and Data Cleaning.* fMRI data were preprocessed using SPM12 (<https://www.fil.ion.ucl.ac.uk/spm/software/spm12/>). DICOM images were converted to 3D NIfTI files, then slice-time corrected using the slice acquired in the middle of the acquisition (slice 1 due to interleaved acquisition). A two-pass procedure for motion correction first realigned all images to the first collected image, and then to the mean of the images after the first realignment. After co-registration to the mean functional image, the anatomical image was segmented into gray matter, white matter, cerebrospinal fluid, bone, soft tissue, and air images. Both normalization and smoothing of the data were performed using the DARTEL algorithm ^5^ , in which a group-level gray matter template was created from the gray and white matter images for each subject and session. The group-level template and functional data were then affine-registered and the functional images were transformed to MNI space using subject- and session-specific flow fields. The normalized fMRI data were then resliced to 2.65 mm^3^ isotropic voxels, and data were smoothed with a 6 mm FWHM Gaussian kernel for univariate fMRI analysis. Data cleaning for the realigned images was performed with ArtRepair Software (<https://cibsr.stanford.edu/tools/human-brain-project/artrepair-software.html>). Individual fMRI volumes with translational movement equaling or exceeding 1 mm, or with mean signal greater than 3 standard deviations above the mean global signal, were flagged for censoring in the general linear models.

*Behavioral Models.* All models included a subjects factor as a random intercept. Models including task factors, such as cue emotion or distractor type, also utilized random intercepts for the interactions of the random effect of subject with the relevant task factor and the study drug intervention, respectively. Fixed effects were tested for significance using Satterthwaite's degrees of freedom method ^6^. Estimated marginal means were calculated for significant fixed effects (“emmeans” and “pairs” functions). Degrees of freedom for these post-hoc tests were estimated using the Kenward-Roger method ^7^.

*fMRI Models.* Within-subject general linear models employed in this study used a high-pass filter of 0.0078 Hz (cutoff period = 128 s) to remove low-frequency drifts in the data, and a first-order autoregressive model to account for serial autocorrelation. Additional regressors of no interest for each subject and model included the six head direction movement parameters, the translational movement per volume estimated by ArtRepair, constant regressors for each run to account for run-specific signal offsets, and stick-function regressors specifying individual volumes for censoring. Each scanning session was estimated separately at the subject-level before being contrasted at the group-level.

Random effects analysis assessing the interaction between task factors was performed on the difference contrasts for tolcapone minus placebo compared between neutral and fearful task conditions, equivalent to the two-factor interaction between these terms *(neutral*tolcapone - neutral*placebo - fearful*tolcapone + fearful*placebo).* The resulting beta values were then regressed against the covariate of interest (i.e. CAPS-5) to examine the three-way interaction. Subsequent brain-behavior relationships between the fMRI contrasts and behavioral scores were performed by taking the Pearson correlation between (a) the average voxel-wise parameter estimate for the 2-way interaction at the decision phase for clusters that covaried significantly with CAPS-5 scores, and (b) the 2-factor interaction for the response bias and sensitivity index measures.

*Significance Testing.* Analysis was performed across the whole-brain as well as within a striatal region of interest encompassing the caudate and putamen. To determine cluster-corrected whole-brain significance, we used the Analysis of Functional NeuroImages (AFNI) software package (<https://afni.nimh.nih.gov/>). To estimate noise smoothness values, the spatial autocorrelation function was derived from the residuals of the first-level general linear models for each subject and imaging session, collapsed across stimulus affect (AFNI function 3dFWHMx). These spatial autocorrelation function estimates were performed separately for the whole-brain mask and striatal region of interest. Significant cluster size thresholds were then estimated by simulating the noise volume using the spatial autocorrelation function estimates and the “NN2” nearest neighbor clustering method (AFNI function 3dClustSim) for the whole brain and the striatal region of interest, respectively. With individual voxel thresholds initially established at p < 0.005 uncorrected, significant clusters were determined to be a minimum of 51 voxels for whole brain analyses, and 13 voxels for the striatal region of interest.

*fMRI Post-Hoc Tests.* Parameter estimates from the univariate and PPI analyses were averaged across significant clusters for each analysis (frontoparietal regions and caudate for the univariate analysis, and default mode regions for the PPI analysis), then entered into R for post-hoc testing. 3-way interactions were recreated using models that included a subjects factor as a random intercept, as well as a random intercept for the interaction of the random effect of subject with the study drug intervention. Similarly to the behavioral analysis, fixed effects were tested for significance using Satterthwaite's degrees of freedom method ^6^, and estimated marginal means were calculated for significant fixed effects (“emmeans” and “pairs” functions). Degrees of freedom for these post-hoc tests were estimated using the Kenward-Roger method ^7^ and p-values were corrected with the Tukey method for comparing a family of 4 estimates (i.e. across PTSD severities) for the 3-way interaction in post-hoc testing.

**Supplementary Results**

*Additional Hypothesis-Driven Behavioral Analyses.* As noted briefly in the main text, a linear mixed model predicting the sensitivity index that included cue emotion (neutral versus fearful), centered PTSD severity, and drug intervention regressors found a significant main effect of cue emotion (F(1, 30.0) = 35.633, p < .0001, adj. R^2^ = 0.5277), an interaction between the drug intervention and centered PTSD severity that approached significance (F(1, 30.0) = 3.760, p = .0620, adj. R^2^ = 0.0818), and a significant interaction between cue emotion and centered PTSD severity (F(1, 30.0) = 8.046, p = .0081, adj. R^2^ = 0.1852). A post hoc paired t-test comparing neutral to fearful faces demonstrated improved performance for neutral faces (t(32.1) = 5.767, p < .0001, d = 2.0358). To examine the cue emotion by PTSD severity interaction, we assessed PTSD severity at one standard deviation below average, average, one standard deviation above average, and two standard deviations above average. Post hoc t-tests were significant for neutral greater than fearful cue emotion at one standard deviation below average PTSD severity (t(32.1) = 6.011, p < .0001, d = 2.1219), average PTSD severity (t(32.1) = 5.767, p < .0001, d = 2.0358), and one standard deviation above average PTSD severity (t(32.1) = 2.128, p = .041, d = 0.7512) but not at two standard deviations above average PTSD severity (t(32.1) = 0.117, p = .9073). That participants with more severe PTSD did not show improved performance for neutral compared to fearful stimuli is consistent with hypotheses that individuals with more severe PTSD may have difficulty differentiating fearful and neutral stimuli ^8^. When PTSD severity was replaced with number of concussions (F(1, 30) = 0.695, p = 0.4111; F(1, 30.0) = 0.229, p = .6357) and working memory span (F(1, 30.0) = 2.113, p = 0.1564; F(1, 30.0) = 1.555, p = .2221), both individually and as interactions, respectively, no findings were significant.

Linear mixed models predicting the sensitivity index and response bias including the emotion of the distractor (neutral vs. arousing) along with the effect of the drug intervention, centered PTSD severity, and the associated interactions produced no significant findings for the distractor emotion factor. The model predicting the sensitivity index did result in a centered PTSD severity by drug intervention predictor (F(1, 30.0) = 5.762, p = .0228, adj. R^2^ = 0.1332), while the model predicting response bias led to drug intervention being a significant predictor (F(1, 30.0) = 5.541, p = .0253, adj. R^2^ = 0.1278), equivalent to effects reported in the main manuscript. Thus, although we did hypothesize that the emotionally-arousing stimuli presented in the distractor phase would impact working memory performance, there was no evidence for this hypothesis from the behavioral analyses in this study. The distractor scene stimuli used in this study were related to subjects’ initial trauma (combat), and the distractor face stimuli expressed fear similar to the faces presented at the cue phase, but it is possible that these images were not sufficiently arousing to evoke emotional activation of schemas related to trauma exposure ^9^. Alternatively, it is possible that the distractor design in our study was conducive to working memory gating mechanisms ^10^ that allowed for less thorough processing of this information along with reduced emotional responding.

*3-way Interaction Post-Hoc Tests.* Findings from the 3-way behavioral interaction discussed in the main text of the paper varied by PTSD severity, and the effects of the drug intervention and cue emotion factors were assessed using estimated marginal means across levels of PTSD severity. At one standard deviation below average PTSD severity, neutral cue faces led to more conservative responding than fearful cue faces on placebo (t(59.9) = 3.600, p = .0035, d = 0.9303), neutral cue faces did not lead to more conservative responding than fearful faces on tolcapone (t(62.6) = 2.560, p = .061, d = 0.6471), neutral cue faces on tolcapone led to more conservative responding than fearful cue faces on placebo (t(62.6) = 4.236, p = .0004, d = 1.0708), and neutral cue faces on tolcapone led to more conservative responding than fearful cue faces on tolcapone (t(59.9) = 4.778, p = .0001, d = 1.2373). At average PTSD severity, neutral cue faces led to more conservative responding than fearful cue faces on placebo (t(59.9) = 6.920, p < .0001, d = 1.7882), neutral cue faces on placebo led to more conservative responding than fearful faces on tolcapone (t(62.6) = 3.530, p = .0043, d = 0.8923), neutral cue faces on tolcapone led to more conservative responding than fearful cue faces at placebo (t(62.6) = 6.898, p < .0001, d = 1.7437), and neutral cue faces led to more conservative responding than fearful cue faces on tolcapone (t(59.9) = 5.950, p < .0001, d = 1.5376). For one standard deviation above average PTSD severity, neutral cue faces led to more conservative responding than fearful cue faces on placebo (t(59.9) = 6.167, p < .0001, d = 1.5936), neutral cue faces led to more conservative responding that approached significance on placebo than fearful cues faces on tolcapone (t(62.6) = 2.422, p = .083, d = 0.6122), neutral cue faces on tolcapone led to more conservative responding than fearful cue faces on placebo (t(62.6) = 5.499, p < .0001, d = 1.390), fearful cue faces on tolcapone led to more conservative responding than fearful cue faces on placebo (t(54.7) = 2.839, p = .031, d = 0.7677), and neutral cue faces led to more conservative responding than fearful cue faces on tolcapone (t(59.9) = 3.609, p = .0034, d = 0.9326). Lastly, for two standard deviations above average PTSD severity, neutral cue faces led to more conservative responding than fearful cue faces on placebo (t(59.9) = 4.706, p = .0001, d = 1.2161), neutral cue faces on tolcapone led to more conservative responding than fearful cue faces on placebo (t(62.6) = 3.872, p = .0015, d = 0.9788), and there was more conservative responding on tolcapone than on placebo for fearful cue faces that neared significance (t(54.7) = 2.566, p = .061, d = 0.6939). All p-values were corrected with the Tukey method for comparing a family of 4 estimates (i.e. across PTSD severities).

*Exploratory Behavioral Analyses.* Behavioral models to assess additional task factors of interest, in conjunction with mean-centered PTSD severity, the drug intervention, and their associated interactions, were used to predict the sensitivity index and response bias metrics. Our model examining the task factor of working memory load (i.e. retention of one versus three faces at cue) along with mean-centered PTSD severity, drug intervention, and their interactions to predict the sensitivity index identified working memory load as a significant predictor (F(1, 30.0) = 119.642, p < .0001, adj. R^2^ = 0.7928) as well as a significant interaction between the drug intervention and PTSD severity (F(1, 30.0) = 6.408, p = .0168, adj. R^2^ = 0.1485). A follow-up post-hoc test showed that performance was significantly better in the low load compared to high load condition (t(32.1) = 10.567, p < .0001, d = 3.7302). The same model used to predict response bias instead of sensitivity index also resulted in working memory load as a significant predictor (F(1, 30.0) = 6.236, p = .01824, adj. R^2^ = 0.1445), as was the drug intervention (F(1, 30.0) = 4.630, p = .0396, adj. R^2^ = 0.1048). A post-hoc test comparing load conditions showed that participants were more conservative in the high load compared to low load condition (t(32.1) = 2.412, p = .022, d = 0.8514).

Next we examined linear mixed models including mean-centered PTSD severity, the drug intervention, and task factors from the distractor phase to predict sensitivity index and response bias, respectively. The model examining the type of distractor (face vs. place) along with PTSD severity, the drug intervention, and the associated interactions to predict sensitivity index revealed a significant main effect of type of distractor (F(1, 60.0 = 12.306, p = .0009, adj. R^2^ = 0.1564) as well as a centered PTSD severity and drug intervention interaction (F(1, 30.0) = 6.808, p = .0140, adj. R^2^ = 0.1578). A post-hoc t-test comparing distractor types showed that participants performed better when the distractor stimulus was a place image instead of a face stimulus (t(32.1) = 3.389, p = .0019, d = 1.1963) – i.e. when the cue and distractor were more distinct, or equivalently, less congruent. When the same model was used to predict response bias, the main effect of distractor type was a significant predictor (F(1, 30.0) = 4.488, p = .0425, adj. R^2^ = 0.1011) along with the drug intervention (F(1, 30.0) = 4.546, p = .0413, adj. R^2^ = 0.1026). The post-hoc test comparing distractor stimuli revealed that participants responded more conservatively to the decision phase of the task when the distractor image was a face instead of a place stimulus (t(32.1) = 2.047, p = .0490, d = 0.7226).

*Control Analyses.* We were interested in whether the number of concussions experienced by the participants could explain the experimental results instead of PTSD severity. For concussions, previous work has argued that a distant history of mild traumatic brain injury (mTBI) does not explain chronic somatic symptoms after the statistical model accounts for the effects of PTSD ^11^, though this finding has been controversial at times ^12^. We do note that traditional classification schemes, such as a division into mild, moderate, and severe TBI based on GCS, duration of post-traumatic amnesia, and loss of consciousness, would not distinguish the subjects here: all of our subjects had mild TBI that occurred more than 1 year prior, so we used the number of concussions to approximate the mTBI burden in our sample. Furthermore, we also investigated working memory span as a control factor of interest. CAPS-5 scores were not significantly correlated with the number of concussions (r = .2709, p = .1476) or the working memory span (r = -.1027, p = .5892), so any significant effects of these factors should be effectively independent of PTSD severity.

To assess the specificity of the interaction between the drug intervention for PTSD severity, we reran the above models for d’ and response bias, replacing PTSD severity with the mean-centered number of concussions and baseline working memory span (obtained at screening using the Daneman & Carpenter listening span ^13^), respectively. For d’, neither the number of concussions (F(1, 30) = 0.561, p = 0.460) and its interaction with the drug intervention (F(1,30) = 2.542, p = 0.1213), nor the baseline working memory span (F(1, 30) = 1.940, p = 0.1739) and its interaction with drug intervention (F(1,30) = 0.014, p = 0.9064), were predictive. For response bias, which did not interact with PTSD severity, additional models also revealed that neither the number of concussions nor the baseline working memory span were significant predictors.

We also performed a similar set of control analyses assessing whether number of concussions or working memory span could explain the 3-way interaction between the drug intervention, cue emotion, and centered PTSD severity. When PTSD severity was replaced with number of concussions, the main effect and interaction were not significant (F(1, 30.0) = 1.587, p = 0.218; F(1, 30.0) = 1.885, p = 0.180). For baseline working memory span, the main effect of working memory span approached significance, but no significant three-way interaction was observed (F(1, 30) = 3.612, p = 0.0670, adj. R^2^ = 0.0777; F(1, 30) = 1.430, p = 0.241).

In order to assess whether concentration difficulties could explain or mediate our results, we examined the attention subscale from the BIS as a measure of concentration problems in our sample. First, we found behaviorally that scores on this subscale were strongly correlated with CAPS-5 scores (r = 0.3993, p = .02883)), so the independent effects of these variables are not assessable in this dataset. As a result, we examined whether attention could mediate the effect of PTSD severity on our neuroimaging data. When we used this BIS subscale to predict activity within the frontoparietal regions (and caudate) displayed in **Figure 3** from the main text, we did find that it was a significant predictor (p = .0104). However, when we regressed the BIS attention subscale and the CAPS-5 scores together to predict the activation in these clusters, the BIS attention subscale became non-significant (p = .1181), while CAPS-5 remained a significant predictor (p = .0028). The indirect path from CAPS-5 🡪 BIS attention subscale 🡪 frontoparietal activity approached significance (p = .0770), indicating some support for attention as a mediator. Altogether, our study was not able to effectively disentangle these variables enough to firmly establish the relationship between PTSD severity and concentration difficulties in our study.

*COMT Genetics.* COMT genotype data was available for 22 subjects. 10 participants were Val/Val, 10 were Val/Met, and 2 were Met/Met. When we performed a median-split based on PTSD severity, we found that the more severe PTSD group had 4 Val/Val, 7 Val/Met, and 1 Met/Met participants, while the low severity group had 6 Val/Val, 3 Val/Met, and 1 Met/Met participants. A Chi-Square test of independence (*X^2^*(2, *N* = 22) = 1.8333, p = .39985) showed that the distribution of COMT genotype did not differ based on PTSD severity. If confirmed, these results also suggest that PTSD dysfunction may be associated with impaired dopaminergic transmission independent of COMT genotype ^14,15^.

*PPI Parameter Interaction with PTSD Severity.* As briefly discussed in the main text, we found an interaction between centered PTSD severity and stimulus emotion (F(1, 60.0) = 16.585, p = .0001, adj. R^2^ = 0.3275). Post-hoc tests were assessed using estimated marginal means for one standard deviation below average, average, and both one and two standard deviations above average PTSD severity. For PTSD subjects one (t(64.3) = 3.470, p = .0009, d = 0.8655) and two (t(64.3) = 3.953, p = .0002, d = 0.9859) standard deviations above average, there was increased PPI functional connectivity for fearful compared to neutral stimuli, while the opposite effect resulted for the subjects one standard deviation below average PTSD severity (t(64.3) = 2.106, p = .0392, d = 0.5253). These findings may indicate an increased interaction of frontoparietal cognitive control regions and brain regions implicated in intrusive trauma imagery in subjects with more severe PTSD symptoms ^16^.

*fMRI Parameter Estimates for 3-Way Interactions.* BOLD responses for each of the significant regions identified in the univariate and PPI contrasts can be found in **Figures S3 and S4**. Please see the main text for further description of findings collapsed across all significant areas.

| **Category** | **Subcategory** | **Mean/Count** | **Range/Percentage** |
| --- | --- | --- | --- |
| Age |  | 35.5 ± 8.3 | 22-49 |
| Gender | Male | 25 | 83.3% |
|  | Female | 5 | 16.7% |
| Ethnicity | Caucasian | 16 | 53.3% |
|  | African American | 5 | 16.7% |
|  | Asian | 4 | 13.3% |
|  | Mixed | 3 | 10.0% |
|  | Native American | 2 | 6.7% |
| Education (Years) ^1^ |  | 14.0 ± 1.6 | 12-18 |
| CAPS-5 |  | 25.4 ± 19.5 | 0-68 |
| Concussions |  | 2.9 ± 2.1 | 1-10 |
| Depression (CESD-R) |  | 18.4 ± 12.7 | 2-51 |
| Reading (WTAR) |  | 104.8 ± 11.2 | 84-125 |
| Listening Span (M3) |  | 33.0 ± 10.8 | 17-58 |
| Attention (BIS) |  | 12.0 ± 3.2 | 7-18 |

^1^ The number of years of education was not available for one subject.

**Table S1**: Subject demographics.

Univariate Analysis Results (Figure 3, Main Text)

| Brain Region | Extent | t-value | x | y | z |
| --- | --- | --- | --- | --- | --- |
|  |  |  |  |  |  |
| Left Frontal Pole | 270 | 4.207 | -29 | 58 | 16 |
| Right Inferior Frontal Gyrus | 111 | 3.913 | 50 | 27 | 21 |
| Left Intraparietal Sulcus | 95 | 3.906 | -32 | -50 | 50 |
| Left Middle Frontal Gyrus | 59 | 3.878 | -40 | 16 | 40 |
| Left Inferior Frontal Gyrus | 57 | 3.601 | -37 | 16 | 27 |
| Right Intraparietal Sulcus | 76 | 3.483 | 40 | -37 | 48 |
|  |  |  |  |  |  |
| Small Volume Correction |  |  |  |  |  |
| Right Caudate Nucleus | 18 | 3.546 | 13 | 19 | 5 |

PPI Analysis Results (Figure 4, Main Text)

| Brain Region | Extent | t-value | x | y | z |
| --- | --- | --- | --- | --- | --- |
|  |  |  |  |  |  |
| *Left Frontal Pole Seed* |  |  |  |  |  |
| Left Inferior Temporal Gyrus | 66 | 3.679 | -45 | -53 | -16 |
|  |  |  |  |  |  |
| *Left Inferior Frontal Gyrus Seed* |  |  |  |  |  |
| Left Angular Gyrus | 63 | 5.166 | -48 | -64 | 27 |
|  |  |  |  |  |  |
| *Left Middle Frontal Gyrus Seed* |  |  |  |  |  |
| Right Posterior Cingulate Cortex | 145 | 5.288 | 3 | -50 | 27 |
| Left Cuneus | 74 | 3.902 | -5 | -69 | 27 |

**Table S2**: Functional MRI Analyses – Cluster Data. All regions are significant at a level of p < 0.05, corrected. Data for the right caudate utilize a small volume correction (see Supplementary Methods).


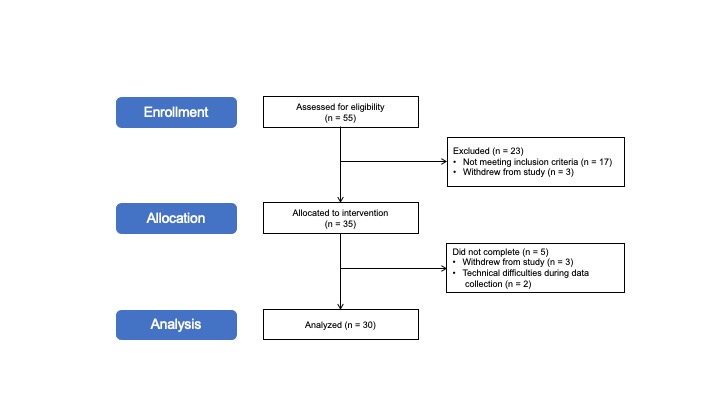
 **Figure S1.** Study participant flow.


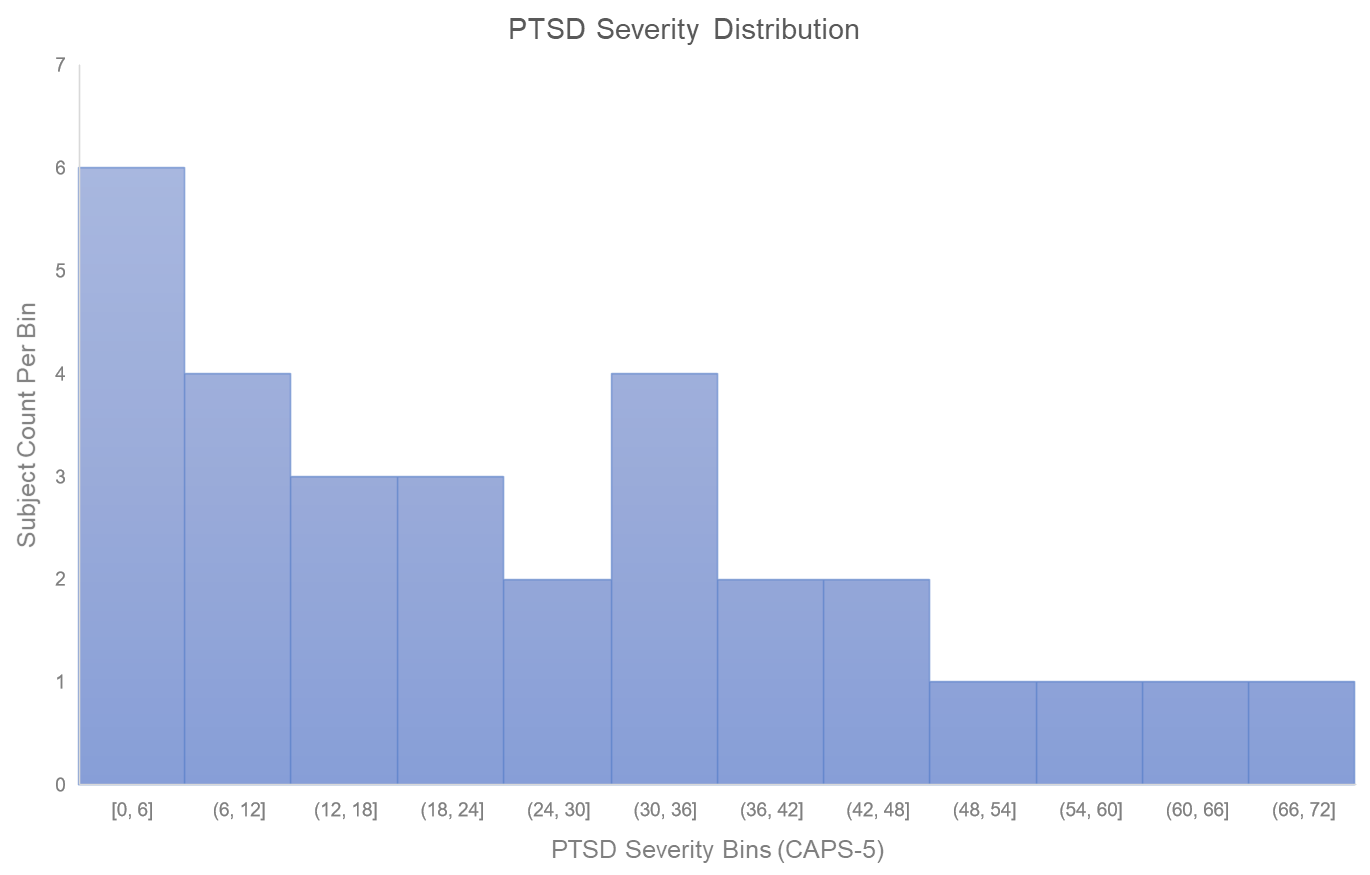


**Figure S2.** Histogram showing the count of participants per PTSD severity bin, measured with the CAPS-5. Square brackets indicate numbers that are included in that bin, while parentheses specify that the number is excluded from the bin.


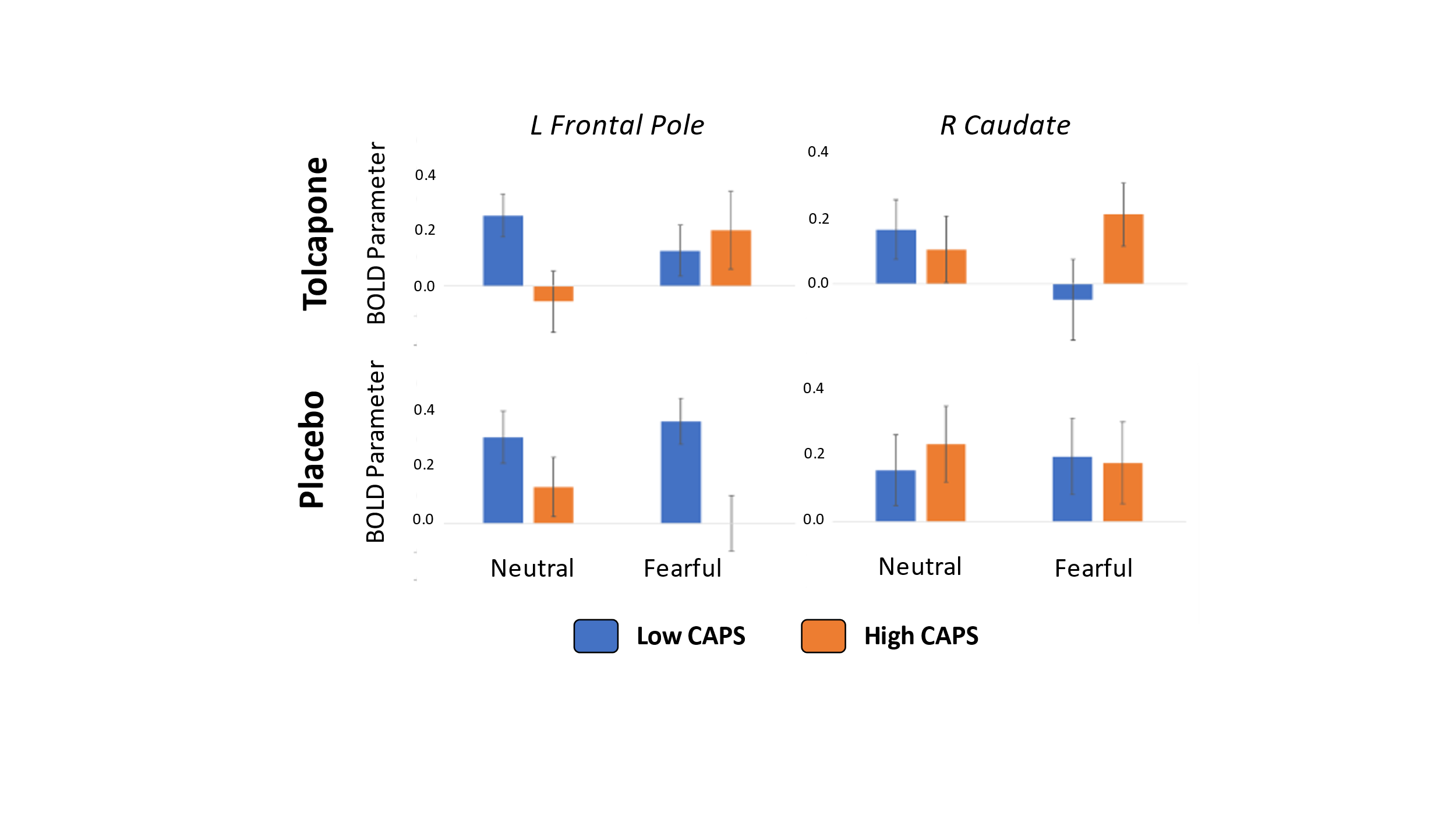


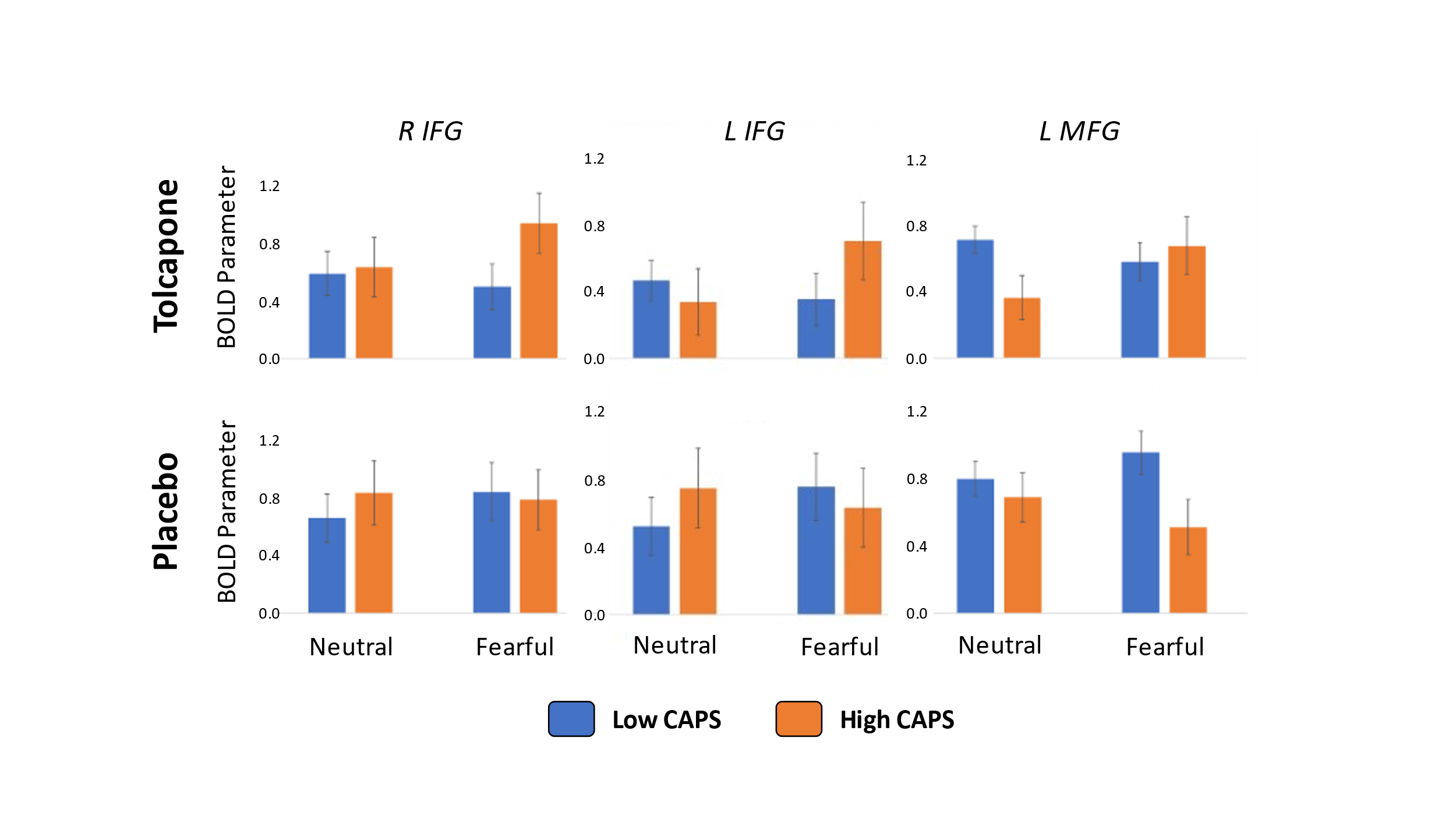


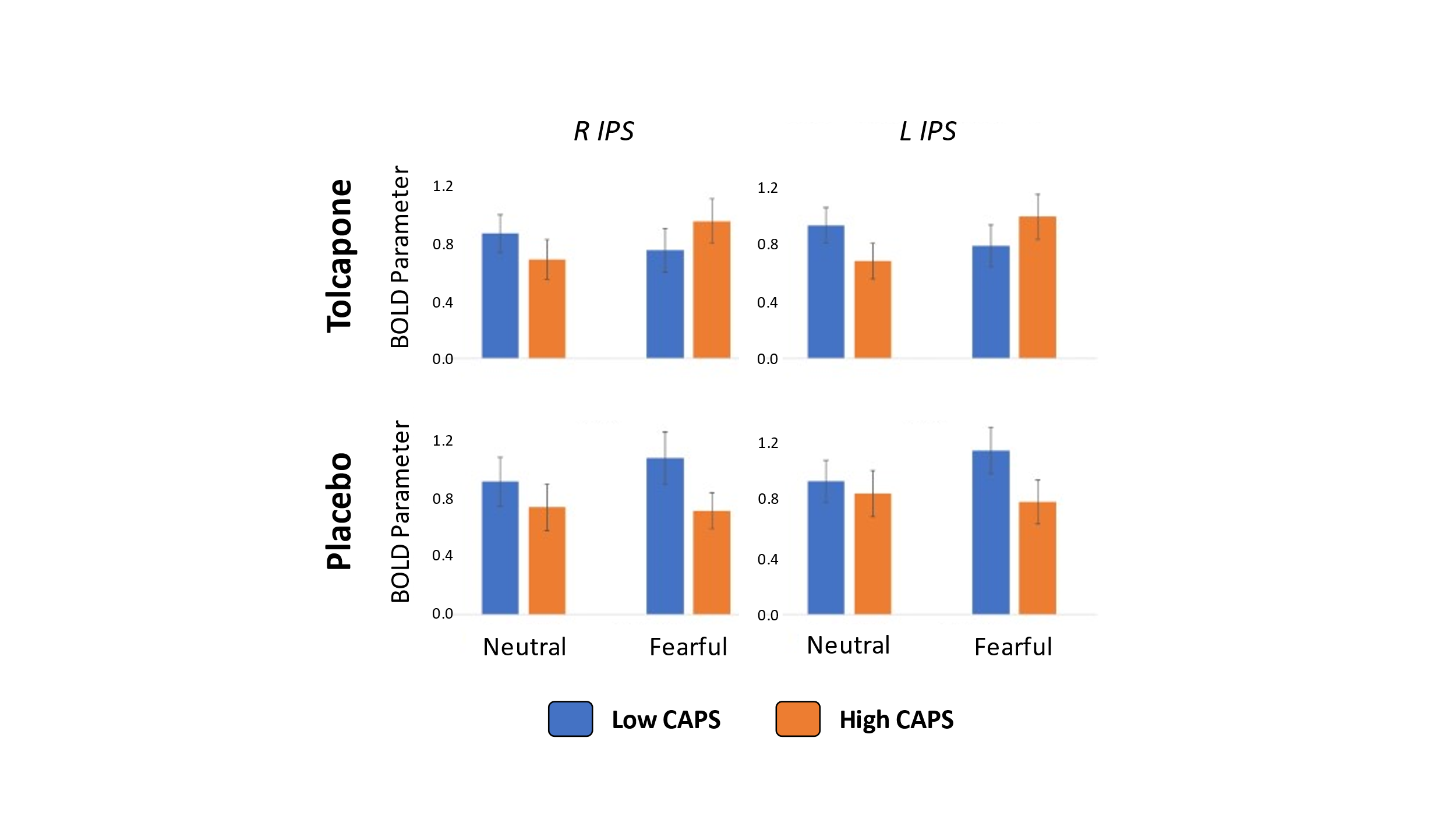


**Figure S3.** BOLD parameter estimates, for significant clusters in the frontal pole and caudate (top row), prefrontal (middle row), and parietal (bottom row) cortex, divided via median split into low CAPS-5 and high CAPS-5 subjects. Abbreviations: IFG = Inferior Frontal Gyrus, MFG = Middle Frontal Gyrus, IPS = Intraparietal Sulcus. See also Figure 3, main text.


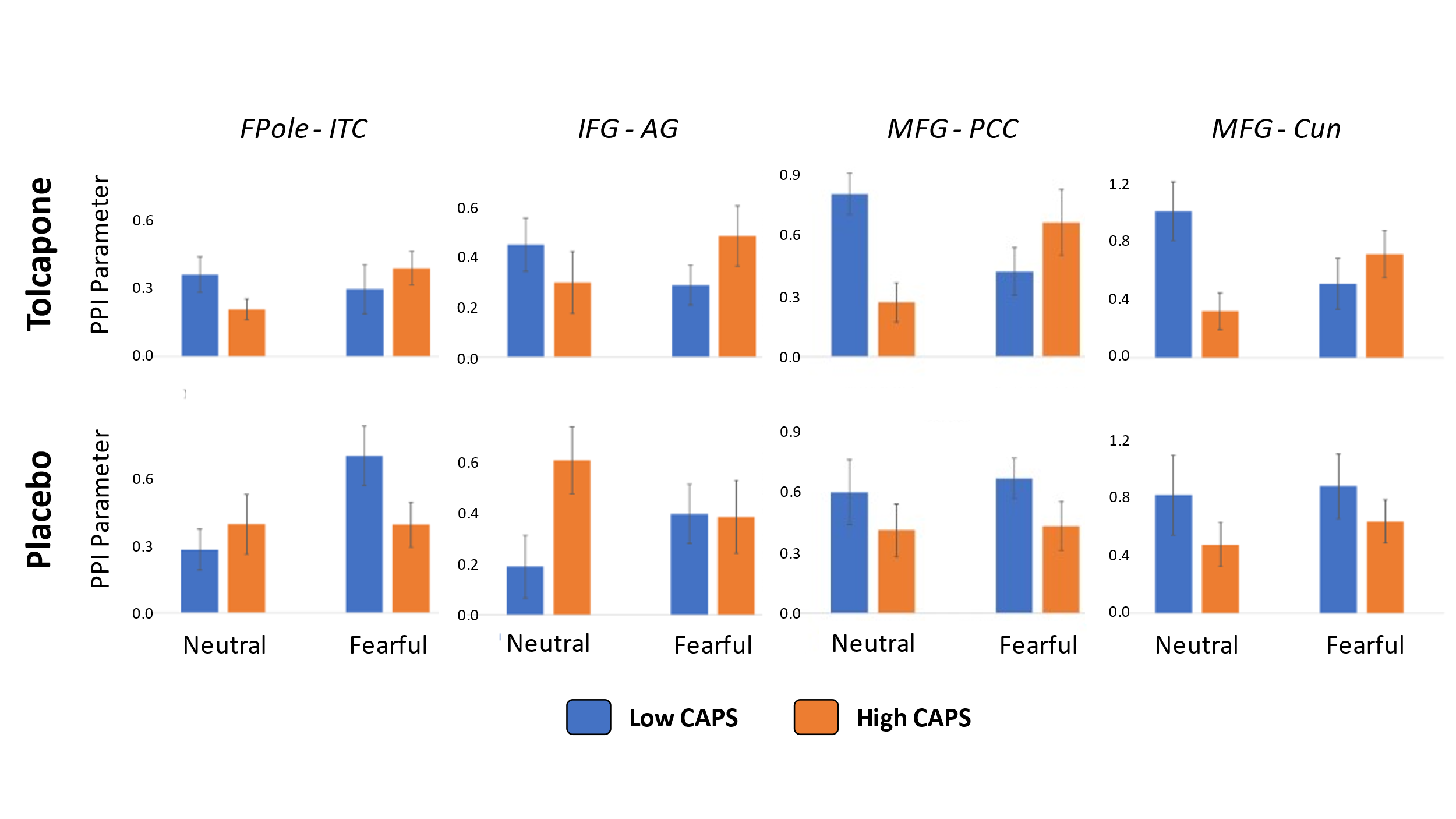


**Figure S4.** PPI parameter estimates, for the significant clusters identified in the psychophysiological interaction (PPI) analysis. Abbreviations: FPole = Frontal Pole, ITC = Inferior Temporal Cortex, IFG = Inferior Frontal Gyrus, AG = Angular Gyrus, MFG = Middle Frontal Gyrus, PCC = Posterior Cingulate Cortex, Cun = Cuneus. See also Figure 4, main text.

**References**

1 Blake, D. D. *et al.* The development of a clinician-administered PTSD scale. *J. Trauma. Stress* **8**, 75-90 (1995).

2 Eaton, W. W., Smith, C., Ybarra, M., Muntaner, C. & Tien, A. Center for Epidemiologic Studies Depression Scale: Review and Revision. *The Use of Psychological Testing for Treatment Planning and Outcomes Assessment: Volume 3: Instruments for Adults*, 363-377 (2004).

3 Stanford, M. S. *et al.* Fifty years of the Barratt Impulsiveness Scale: An update and review. *Pers. Indiv. Differ.* **47**, 385-395 (2009).

4 Green, R. E. A. *et al.* Measuring premorbid IQ in traumatic brain injury: an examination of the validity of the Wechsler Test of Adult Reading (WTAR). *J. Clin. Exp. Neuropsyc.* **30**, 163-172 (2008).

5 Ashburner, J. A fast diffeomorphic image registration algorithm. *Neuroimage* **38**, 95-113 (2007).

6 Kuznetsova, A., Brockhoff, P. B. & Christensen, R. H. B. lmerTest package: tests in linear mixed effects models. *J. Stat. Softw.* **82** (2017).

7 Halekoh, U. & Højsgaard, S. A kenward-roger approximation and parametric bootstrap methods for tests in linear mixed models–the R package pbkrtest. *J. Stat. Softw.* **59**, 1-30 (2014).

8 Besnard, A. & Sahay, A. Adult hippocampal neurogenesis, fear generalization, and stress. *Neuropsychopharmacol.* **41**, 24-44 (2016).

9 Dutra, L., Callahan, K., Forman, E., Mendelsohn, M. & Herman, J. Core schemas and suicidality in a chronically traumatized population. *J. Nerv. Ment. Dis.* **196**, 71-74 (2008).

10 Chatham, C. H. & Badre, D. Multiple gates on working memory. *Curr. Opin. Behav. Sci.* **1**, 23-31 (2015).

11 Hoge, C. W. *et al.* Mild traumatic brain injury in US soldiers returning from Iraq. *New Engl. J. Med.* **358**, 453-463 (2008).

12 Hendrickson, R. C., Schindler, A. G. & Pagulayan, K. F. Untangling PTSD and TBI: Challenges and strategies in clinical care and research. *Curr. Neurol. Neurosci.* **18**, 1-12 (2018).

13 Daneman, M. & Carpenter, P. A. Individual differences in working memory and reading. *J. Verb. Learn. Verb. Be.* **19**, 450-466 (1980).

14 Papalini, S., Beckers, T. & Vervliet, B. Dopamine: from prediction error to psychotherapy. *Transl. Psychiat.* **10**, 1-13 (2020).

15 Torrisi, S. A., Leggio, G. M., Drago, F. & Salomone, S. Therapeutic challenges of post-traumatic stress disorder: focus on the dopaminergic system. *Front. Pharmacol.* **10**, 404 (2019).

16 Sartory, G. *et al.* In search of the trauma memory: a meta-analysis of functional neuroimaging studies of symptom provocation in posttraumatic stress disorder (PTSD). *PloS one* **8**, e58150 (2013).
